# Calcaneal Fracture-Dislocation: A Case Report

**DOI:** 10.7759/cureus.100707

**Published:** 2026-01-03

**Authors:** Ahmad Almigdad, Asmaa Hayadreh, Ehab Altaani, Mohammad Al-Dweeri, Khalid Bani Melhem

**Affiliations:** 1 Department of Orthopedics, Royal Medical Services, Amman, JOR; 2 Department of Family Medicine, Ministry of Health, Amman, JOR

**Keywords:** ankle and foot, calcaneal fracture-dislocation, calcaneus, medial approach, tarsal fractures

## Abstract

Calcaneal fracture-dislocations are rare, high-energy injuries in which the sustentacular fragment typically remains stable, while the posterolateral fragment is displaced laterally. These injuries are frequently associated with additional fractures, ligamentous disruption, or tendon entrapment, and they present considerable diagnostic and surgical challenges. Accurate imaging and meticulous operative planning are essential to restoring hindfoot alignment and function. We report the case of a 45-year-old woman with a calcaneal fracture-dislocation, treated via a medial approach to enable accurate fixation of the posterolateral fragment, potentially reducing the risk of hardware-related tendon irritation.

## Introduction

Calcaneal fractures are the most common tarsal bone fractures, accounting for approximately 60% of tarsal injuries, and are typically caused by high-energy axial loading forces [1]. However, calcaneal fracture-dislocations are uncommon and differ from typical intra-articular calcaneal fractures due to the distinctive displacement pattern of the fracture fragments. While the sustentacular fragment usually remains anatomically aligned with the talus, the posterolateral fragment is laterally displaced, potentially impinging on the talofibular joint or abutting the distal fibula. This displacement pattern is influenced by the inherent structural stability of the calcaneus and the robust ligamentous complexes anchoring it to the talus and cuboid [2]. Unlike conventional subtalar dislocations, these injuries involve a fracture in which the sustentacular fragment remains aligned, while the posterolateral fragment is displaced laterally [3]. Biomechanically, displacement of the posterolateral fragment disrupts subtalar joint congruity and can compromise hindfoot stability, increasing the risk of malunion, post-traumatic subtalar arthritis, and chronic pain if not promptly recognized and managed [4]. Therefore, early recognition and timely intervention are essential to prevent these long-term complications. We report a rare case of calcaneal fracture-dislocation in a 45-year-old female, treated with open reduction and fixation via a medial approach. This case highlights the importance of CT imaging for accurate assessment, as plain radiographs may fail to detect such injuries.

## Case presentation

Patient history and presentation

**Figure 1:**
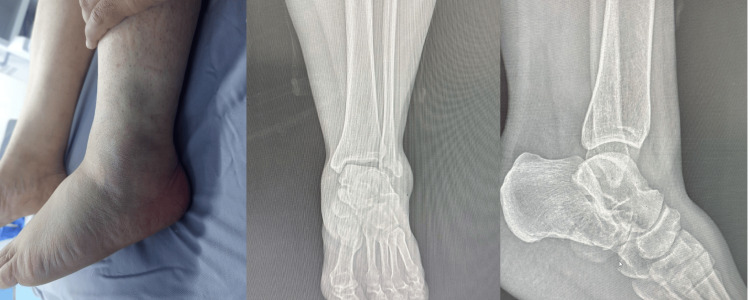
Ankle swelling with lateral ecchymosis; anteroposterior and lateral ankle radiographs. Clinical photograph showing swelling of the ankle and foot with lateral ankle ecchymosis (left). Anteroposterior ankle radiograph demonstrating a distal fibular tip fracture with associated talar tilt (middle). Lateral ankle radiograph showing the “double-density” sign, indicative of overlap of the lateral calcaneal wall (right). However, diagnosing calcaneal fracture-dislocation on plain radiographs can be difficult and requires a high index of suspicion, with CT imaging needed for confirmation.

A 45-year-old woman with no known medical conditions sustained a fall from a height of approximately half a meter, landing on her left ankle. She presented to the emergency department with ankle pain, swelling, and inability to bear weight, with no other associated injuries. Examination revealed diffuse circumferential ankle swelling and ecchymosis over the lateral aspect (Figure 1). Neurovascular status was intact.

Imaging studies

**Figure 2:**
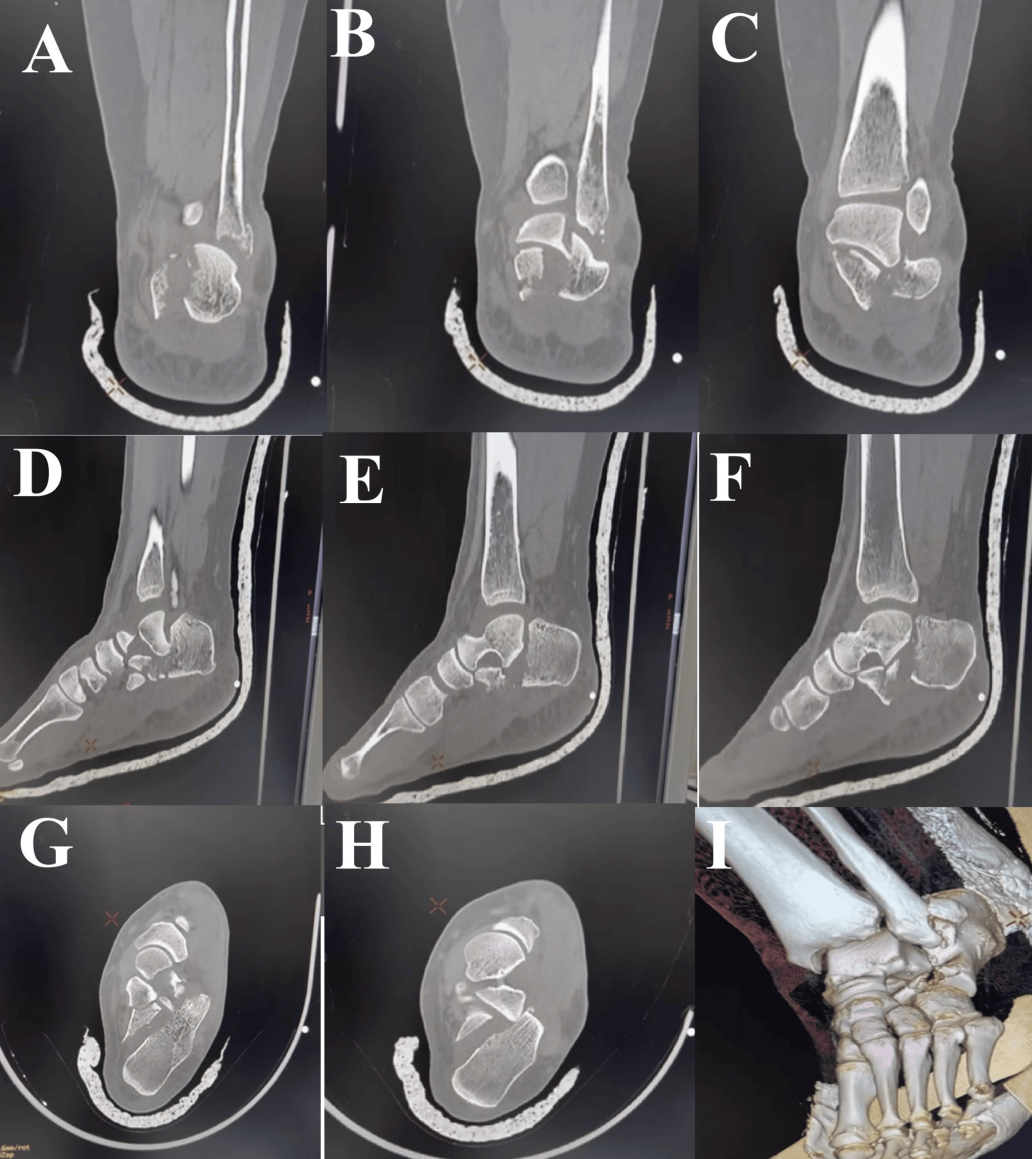
CT imaging of the ankle demonstrating a calcaneal fracture. (A-C) Coronal CT images show a comminuted intra-articular calcaneal fracture with lateral displacement of the posterolateral fragment and a nondisplaced sustentacular fragment. A concomitant fibular tip fracture is also visible. (D-F) Sagittal CT views reveal loss of calcaneal height, involvement of the posterior facet, and lateral extrusion of the posterolateral fragment. (G-H) Axial CT cuts highlight marked lateral translation and rotation of the posterolateral fragment relative to the subtalar joint, with no dislocation of the sustentaculum tali. (I) Three-dimensional reconstruction illustrates the overall morphology of the fracture-dislocation and the spatial relationship between the displaced fragment, the sustentaculum tali, and the distal fibula.

Initial ankle radiographs suggested a distal fibular tip fracture with associated talar tilt (Figure 1). Because calcaneal fracture-dislocations may be difficult to identify on plain radiographs, computed tomography was obtained and confirmed a comminuted calcaneal fracture with displacement of the posterolateral fragment (Figure 2). A below-knee cast was applied, and the patient was admitted for limb elevation and observation.

Surgical procedure

**Figure 3:**
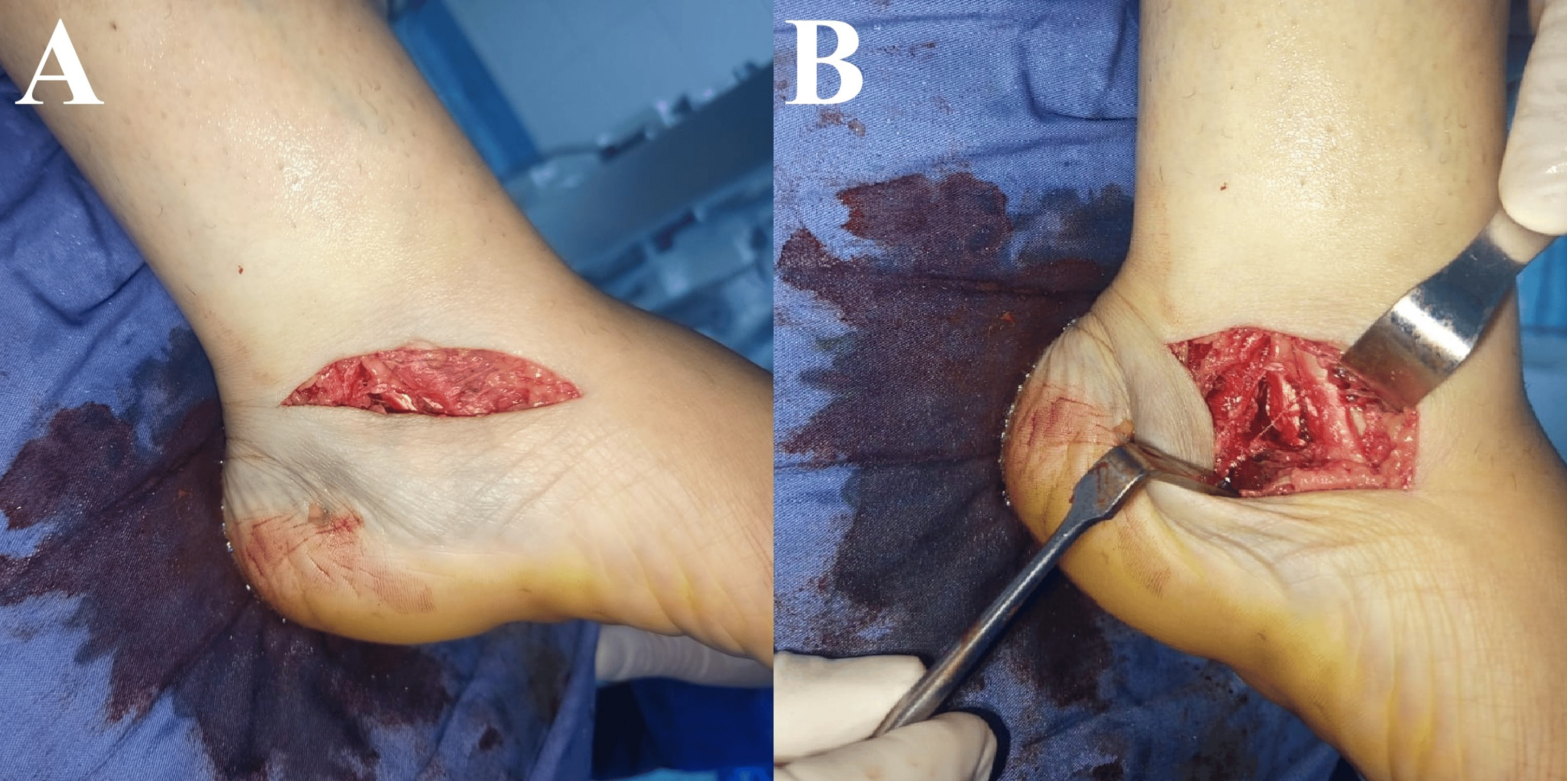
Clinical intraoperative photographs demonstrating the medial approach to the calcaneus. (A) Medial skin incision extending from just posterior to the medial malleolus toward the navicular. (B) Deeper exposure revealing the fracture site, with the surgical interval created between the posterior tibial nerve and the flexor hallucis longus tendon.

For precise screw placement into the sustentaculum tali, a medial approach to the calcaneus was selected, although the sinus tarsi approach can provide easier access to the dislocated posterolateral fragment. The procedure was performed under spinal anesthesia with tourniquet control. A 5-cm incision was made along the course of the neurovascular bundle, extending from just posterior to the medial malleolus toward the navicular (Figure 3). After the skin incision, the neurovascular structures were identified, and dissection was carried out between the posterior tibial nerve and the flexor hallucis longus tendon. Deep dissection beneath the flexor hallucis longus exposed the sustentaculum tali and the medial wall of the calcaneus.

**Figure 4:**
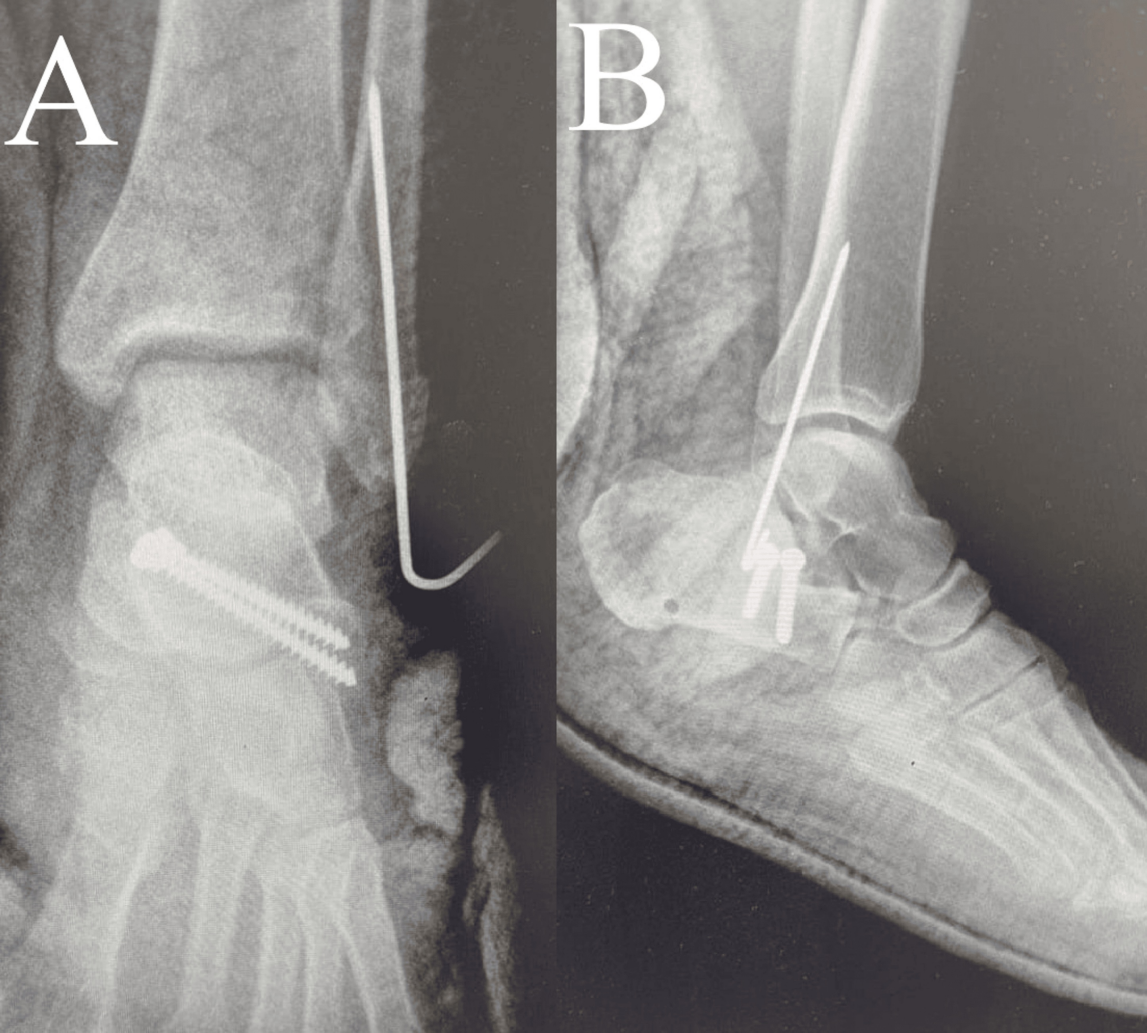
Postoperative images of the ankle. (A) Anteroposterior view and (B) lateral view demonstrating anatomic reduction of the fracture and restoration of subtalar joint alignment.

A Steinmann pin was inserted through the calcaneal tuberosity to assist with fracture reduction. Two 3.5-mm screws were placed from the sustentaculum tali into the posterolateral fragment, and a K-wire was inserted percutaneously to stabilize the fibular tip fracture (Figure 4). The wound was then closed and dressed, and a below-knee cast was applied.

## Discussion

Fracture-dislocations of the calcaneus are exceptionally rare injuries, with only small case series documented since the condition was first described in 1936 [5]. Most published reports [6-9] involve lateral fracture-dislocations, typically presented as isolated cases or small cohorts. A single case of medial luxation has been reported by Anglen and Gehrke [8], attributed to interposition of the flexor hallucis longus tendon. Despite the accumulated literature, no standardized classification system has been established to assist in diagnosis or management, and the true prevalence of this injury pattern remains likely underrecognized.

Calcaneal fracture-dislocations most commonly involve Sanders type II or III fractures. These injuries typically result from high-energy axial loading on an inverted foot, which drives the posterolateral edge of the talus downward and splits the calcaneus into anteromedial and posterolateral fragments. Continued force may disrupt supporting ligaments and cause the posterolateral fragment to dislocate laterally, sometimes accompanied by talar or fibular fractures [1]. Miller and Kwon [9] reported a variants that include the “joint-elevation” pattern in which part of the posterior facet is elevated above the talus, similar to our case. They are often accompanied by associated injuries, including talar or fibular fractures, lateral ligament disruption, tendon dislocation, or flexor hallucis longus entrapment.

Early detection of calcaneal fracture-dislocations is crucial. Patients often show hindfoot swelling, deformities, displaced tendons, or, rarely, checkrein deformity. Neurovascular involvement may occur, but there is currently no standardized classification to guide diagnosis or prognosis [3]. While plain radiographs may reveal signs such as varus tilt of the talus, distal fibular fractures, a wedged fragment in the talofibular joint, or the “double-density” sign indicating lateral wall overlap [10], they can also miss the diagnosis due to atypical radiographic signs, such as the absence of the double-density sign or varus tilt. Additionally, the small size of the dislocated fragment can obscure typical findings, and limited recognition by physicians can lead to confusion with more common injuries like subtalar dislocations. Together, these factors complicate the accurate diagnosis of calcaneal fracture-dislocations on plain radiographs [4].

Surgical intervention is generally recommended for calcaneal fracture-dislocations because conservative management is associated with suboptimal outcomes. Common surgical approaches include open reduction and internal fixation (ORIF), mini-open techniques, percutaneous reduction, and external fixation. The lateral approach is frequently preferred due to superior visualization of fracture fragments and joint surfaces. In patients with high surgical risk or comorbidities, minimally invasive techniques may be considered to reduce soft tissue complications. However, existing studies vary in reporting outcomes, and there is limited consensus on the optimal technique, highlighting a need for further comparative research to establish evidence-based guidelines [9].

The medial approach allows direct visualization of the sustentaculum tali and posteromedial fragments, but it carries potential risks. These include neurovascular injury, particularly to the posterior tibial artery and tibial nerve, tendon irritation or damage, most commonly affecting the tibialis posterior, flexor digitorum longus, and flexor hallucis longus, and wound healing issues due to the limited medial soft tissue coverage. To reduce these risks, careful dissection, meticulous handling of soft tissues, and appropriate screw placement, ideally from medial to lateral to prevent tendon irritation, are essential. Recognizing these potential complications is crucial for achieving stable fixation while protecting surrounding anatomical structures [11,12]. In our case, we opted for a medial approach, as we believed it would provide more stable fixation of the sustentaculum tali to the posterolateral fragment with optimal screw placement and length. Fixation from lateral to medial may not always allow secure purchase or ideal screw positioning, particularly depending on intraoperative fluoroscopic guidance. As this report involves a single patient, we cannot establish general guidelines or recommendations; however, we considered the medial approach to be the most suitable option for this particular case.

## Conclusions

Calcaneal fracture-dislocations are rare, complex injuries that require careful clinical and radiologic evaluation. CT imaging is crucial for accurately assessing fragment displacement, as plain radiographs may be insufficient. The medial approach may allow optimal screw placement in the sustentaculum tali, minimizing the risk of tendon irritation associated with long screws placed from the lateral wall. Importantly, no standardized classification exists for these injuries, underscoring the need for further research to guide their management and outcomes.
